# Oral Administration of Human Polyvalent IgG by Mouthwash as an Adjunctive Treatment of Chronic Oral Candidiasis

**DOI:** 10.3389/fimmu.2018.02956

**Published:** 2018-12-21

**Authors:** Sigifredo Pedraza-Sánchez, Julia I. Méndez-León, Yolanda Gonzalez, María Laura Ventura-Ayala, María Teresa Herrera, Jose Luis Lezana-Fernández, Joseph A. Bellanti, Martha Torres

**Affiliations:** ^1^Unidad de Bioquímica, Instituto Nacional de Ciencias Médicas y Nutrición Salvador Zubirán, Mexico City, Mexico; ^2^Centro de Inmunología Avanzada, Hospital Angeles Lomas, Mexico City, Mexico; ^3^Departamento de Investigación en Microbiología, Instituto Nacional de Enfermedades Respiratorias Ismael Cosío Villegas, Mexico City, Mexico; ^4^Laboratorio de Fisiología Pulmonar, Hospital Infantil de México Federico Gómez, Mexico City, Mexico; ^5^Department of Pediatrics and Microbiology-Immunology, Georgetown University Medical Center, Washington, DC, United States

**Keywords:** *Candida albicans*, phagocytosis, opsonization, NADPH oxidase, mouthwash, human immunoglobulin, mucocutaneous candidiasis, immunodeficiency

## Abstract

*Candida albicans* is a commensal fungus that can cause disease ranging in severity from moderate to severe mucosal infections to more serious life-threating disseminated infections in severely immunocompromised hosts. Chronic mucocutaneous candidiasis (CMC) occurs in patients with mutations in genes affecting IL-17-mediated immunity, such as *STAT3, AIRE, RORC, CARD9, IL12B*, and *IL12RB1*, or gain of function (GOF) mutations in *STAT1*. New strategies for the treatment of candidiasis are needed because of the increased burden of infections and the emergence of drug-resistant strains. In this study, we investigated an aspect of the role of antibodies in the control of *C. albicans* infection. We tested *in vitro* the effects of *C. albicans* opsonization with commercial human polyvalent intravenous IgG (IV IgG) on NADPH oxidase activity and killing of the fungi by blood leukocytes from 11 healthy donors and found a significant enhancement in both phenomena that was improved by IV IgG opsonization. Then, we hypothesized that the opsonization of *Candida in vivo* could help its elimination by mucosal phagocytes in human patients with mucocutaneous candidiasis. We tested a novel adjunctive treatment for oral candidiasis in humans based on topical treatment with IV IgG. For this purpose, we choose two pediatric patients with well-characterized primary immunodeficiencies who are susceptible to CMC. Two 8-year-old female patients with an autosomal recessive mutation in the *IL12RB1* gene (P1, with oral candidiasis) and a GOF mutation in *STAT1* (P2, with severe CMC persistent since the age of 8 months and resistant to pharmacological treatments) were treated with IV IgG administered daily three times a day as a mouthwash over the course of 2 weeks. The treatment with the IV IgG mouthwash reduced *C. albicans* mouth infection by 98 and 70% in P1 and P2, respectively, after 13 days, and complete fungal clearance was observed after complementary nystatin and caspofungin treatments, respectively. Therefore, treatment of oral candidiasis with human polyvalent IgG administered as a mouthwash helps eliminate mucosal infection in humans, circumventing drug resistance, and opening its potential use in patients with primary or transient immunodeficiency.

## Introduction

Candidiasis is a fungal infection caused by yeasts belonging to the genus *Candida*. Over 20 species of Candida yeasts can cause infection in humans, and the most common species is *Candida albicans*, which is a commensal organism that can be isolated from oral mucosa, the intestinal tract, and vaginal mucosa in healthy individuals. Therefore, the presence of this fungus is mostly harmless, but in hosts with an immunodeficiency, this fungus can be pathogenic, causing disease ranging in severity from moderate to severe mucosal infections to more serious life-threating disseminated infections in severely immunocompromised hosts (1). Thus, the susceptibility to Candida infections, including species other than *C. albicans*, is strongly related to certain types of primary immunodeficiencies, immunodeficiencies secondary to immunosuppressive medical interventions, or other infections, such as HIV (2). Recently, the increasing global epidemiological burden of candidal infections and the emergence of drug-resistant strains have prompted an intensive search for new strategies of treatment (3, 4).

The immune response to *Candida* involves the participation of both the innate and adaptive branches of the immune system. The innate immune response occurs by pattern recognition receptors, such as macrophage mannose receptor, TLRs, DC- SIGN, Galectin, and Dectin 1 and 2, which target fungal cell wall components, such as β-glucan or α mannan; this response also involves phagocytosis by macrophages and neutrophils, which produces toxic oxygen and nitrogen metabolites, antimicrobial peptides, and NETs (Neutrophil extracellular traps formed by proteins, DNA, and the antimicrobial peptide calprotectin) (2, 5). The contribution of the adaptive immune response to *Candida* includes the participation of Th1 and Th17 cells. Distinct evidence obtained from studies involving animal models and humans suggests that the Th17 immune response plays a major role in the control of mucosal *Candidal* infections [reviewed in (6, 7)]. Th2 cells, which cooperate with B cells in antibody production, are also activated in response to *Candida* infection, and the antibodies produced against *Candida* can not only neutralize antigens, including virulence factors, and adhesion molecules, that interfere with cell adherence and colonization but also promote fungal opsonization, phagocytosis, killing, and complement activation and participate in antibody-dependent cellular toxicity (8–11). Consequently, the antibodies produced against *Candida* play an important role in the inhibition of the dissemination of infections (11–13) as recently observed in patients with candidemia (14).

Phagocytosis is a cellular mechanism performed by professional phagocytic cells, i.e., dendritic cells, neutrophils, monocytes, and macrophages, whose function is to eliminate invading microorganisms that breach the epithelial barriers. Complement components C3a and C5b and immunoglobulins (e.g., IgG) bind the surface of microorganisms, where they are recognized by complement or IgG Fc receptors on the membrane of neutrophils and macrophages to facilitate phagocytosis and improve intracellular killing, mainly by mechanisms dependent on nitrogen, or oxygen metabolites (15). An *in vitro* study reported by Lehrer and Cline (16) showed that human blood cells enhanced with serum antibodies from healthy AB positive individuals could help eliminate or limit *C. albicans* growth by phagocytes by mechanisms independent of complement and dependent on hydrogen peroxide. *C. albicans* can be killed intracellularly by human neutrophils either through antibody-dependent or antibody-independent mechanisms that utilize NADPH oxidase or CARD9-activation pathways, respectively (17).

In the present study, we first examined whether the opsonization of *C. albicans* with commercial intravenous human polyvalent immunoglobulin (IV IgG) or immune human serum could improve NADPH oxidase activity and the killing of the fungus by blood leukocytes from healthy donors. Based on the positive results obtained in these *in vitro* experiments, we hypothesized that the local application of gamma globulin in the oral cavity of patients with oral candidiasis could facilitate the *in vivo* opsonization and killing of Candida and help the elimination of the fungi. We tested our hypothesis by giving mouthwash treatments with IV IgG preparations to two patients with primary immunodeficiency suffering from chronic mucocutaneous candidiasis. Our results showed that the *in vivo* topical oral gamma globulin treatment helped *Candida* elimination.

## Materials and Methods

### *C. albicans* Culture, Killing, and Opsonization

*C. albicans* (ATCC 14053) was grown in liquid media brain heart infusion, BHI (Oxoid - Thermo Fisher, Waltham, MA, U.S.A.), harvested and stored in frozen aliquots at −70°C. For the NADPH oxidase experiments*, C. albicans* was heat killed (Hk) by incubating an RPMI-yeast suspension (26 × 10^6^ yeast cells) in a water bath at 65°C for 60 min; the failure of fungal growth on Sabouraud agar was used to validate the lack of viability after the heat killing. After performing the preliminary titration experiments, 6 × 10^6^ Hk *C. albicans* yeast were placed in microfuge tubes, opsonized with 0, 1.5, or 15 mg/mL of human polyvalent IgG (IV IgG, Sandoglobulin CSL Behring, PA, USA) or 3 or 30% of human immune serum (HIS, which was obtained from a patient with recurrent empyema with *Candida* and *Klebsiella* infections) for 30 min at 37°C, and immediately transferred to an ice bath. For the killing experiments, live *C. albicans* were opsonized by incubating 6.5 × 10^6^ yeast cells with IV IgG at 15 mg/mL or 30% of HIS for 30 min at 37°C and 5% CO_2_.

### *In vitro* Assessment of NADPH Oxidase Activity in Blood Leukocytes Stimulated With *C. albicans*

The NADPH oxidase activity in blood leukocytes from 11 healthy adult (6 women and 5 men) volunteers aged 26–45 years (median 33 years), who provided informed written consent, was assessed by flow cytometry after oxidation of dihydrorhodamine (DHR) 123 to rhodamine, which was performed using previously described methodology with some modifications (18). Briefly, the blood leukocytes were incubated with Hk *C. albicans* as follows: for each condition tested, 120 μL of venous blood were mixed with 4 mL of cold lysis buffer (NH_4_ Cl 155 mM, NaHCO_3_ 14 mM, and EDTA 0.13 mM) in 15-mL conical polypropylene tubes and incubated for 20 min in an ice bath. After centrifugation for 10 min at 400 × g at 4°C, the supernatants were discarded. The leukocyte pellets at the bottom of the centrifuge tubes were gently resuspended, washed again with cold PBS, and finally resuspended in 100 μL of plain RPMI media. Each tube was gently mixed with a micropipette with 50 μL (3 × 10^5^ yeast) of Hk *C. albicans* (with or without opsonization) and incubated at 37°C and 5% CO_2_ for 1 h. A positive methodologic control for the NADPH oxidase activation of leukocytes was included in each experiment by including specimens stimulated with 70 ng of PMA (Sigma Aldrich, St. Louis, MO, U.S.A.) for 30 min. Then, without washing, 1 μg of DHR 123 (stock dissolved in DMSO and then diluted in PBS) (Sigma Aldrich) was added to each tube, followed by an additional 30 min of incubation at 37°C. The tubes were washed with 5 mL of cold PBS and centrifuged at 400 × g for 7 min; then, the supernatants were decanted, and the leukocyte pellets were resuspended in 500 μL of cold PBS and transferred to flow cytometry tubes. The cells were harvested and analyzed using a FACSCalibur flow cytometer (Becton Dickinson, San Jose, CA, U.S.A.). In total, 10^7^ events were acquired and stored per tube and condition, and the results were analyzed by the mean fluorescence intensity (MFI) of rhodamine gating on granulocytes. The stimulation indices per donor and condition were calculated by dividing the rhodamine MFI with opsonized *Candida* by rhodamine MFI with unopsonized *Candida*. The statistical analyses were performed using the Wilcoxon test with Prism software (GraphPad software, La Jolla, CA, U.S.A.) to compare the indices of NADPH oxidase activity measured under different opsonization conditions. Statistical significance was considered at *p* < 0.05.

### *C. albicans* Killing by Blood Leukocytes From Healthy Donors in a Whole Blood Assay

Subsequently, we evaluated the capacity of blood leukocytes to kill ounopsonized and opsonized *C. albicans* in experiments using whole blood from healthy adult volunteers based on a previously published method (19) with some modifications. Briefly, heparinized blood samples were placed on a rocker shaker to maintain the blood motility and preserve the cell viability. In total, 300 μL of whole blood were placed in 2-mL plastic vials, inoculated with 300 μL of *C. albicans* suspension (3 × 10^3^ *C. albicans* yeasts with or without opsonization) and incubated at 37°C and 5% CO_2_ for 1 h under gentle shaking in a rocker shaker. The infected blood samples were centrifuged at 11,750 × g for 10 min in a microcentrifuge, and the supernatants were discarded. The cells were lysed by adding 1 mL of distilled sterile water and vigorously shaken for 1 min, followed by centrifugation at 11,750 × g for 10 min, and the supernatant was discarded. The *Candida* pellets were resuspended in liquid media BHI, 10 μL of each serial dilution (10^−1^-10^−5^) were seeded in triplicate on Sabouraud agar, and the CFU was determined after a 24-h after incubation at 37°C and 5% CO_2_. The killing of *C. albicans* was estimated on the basis of the enumeration of the CFUs. The statistical analysis was performed by the Wilcoxon test with Prism software.

### Patients With Primary Immunodeficiency Treated for Oral Candidiasis With Topical IV IgG

**Figure 1:**
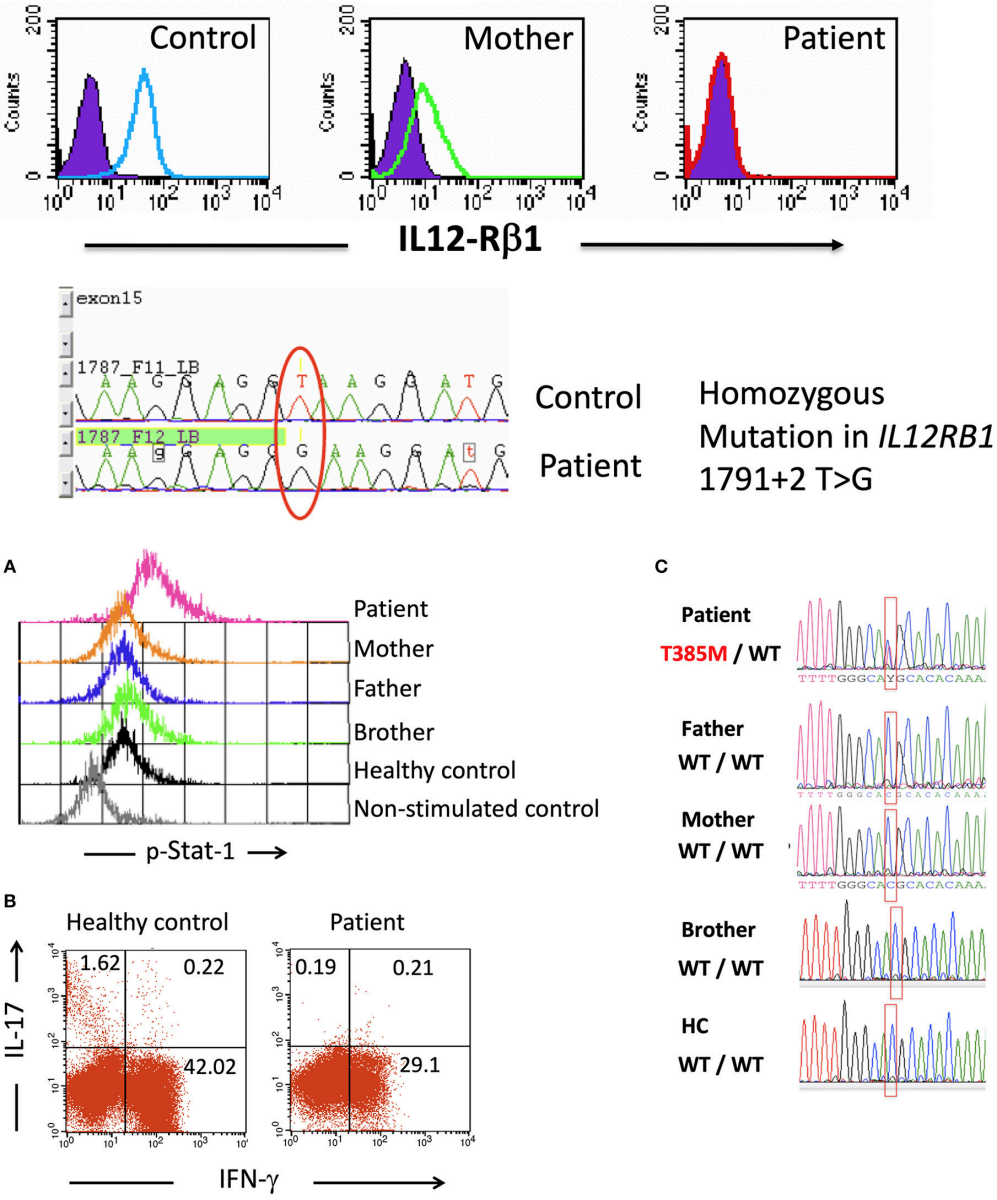
Upper: IL-12Rβ1 expression on day 3 PHA-T blasts in cells from a healthy control, the mother of patient 1, and patient 1 as assessed by flow cytometry (filled histograms, isotype internal control, and open histograms, β1 chain of IL-12 receptor). Compared to a healthy control, the DNA sequencing showed a mutation at exon 15—intron 15–16 in the samples from the patient. Lower **(A)** Intracellular p-Stat1 in monocytes from patient 2 and controls assessed by flow cytometry showing Stat1 hyperphosphorylation in monocytes from the patient (peripheral blood mononuclear cells, PBMCs, were stimulated with recombinant human IFN-γ for 30 min; then, the membranes were labeled with anti-CD14+, and the cells were intracellularly stained for p-Stat1). **(B)** Intracellular production of IFN-γ and IL-17 in CD3+ blood cells from a control and patient 2. PBMCs were stimulated with PMA + ionomycin for 6 h in the presence of Brefeldin A, labeled on membranes with anti-CD3 PerCP and intracellularly stained with anti-IFN-γ Alexa Fluor 488 and anti-IL-17 PE. The numbers shown in the quadrants represent the percentages of positive cells. **(C)** Sequencing of the *STAT1* gene showed the heterozygous mutation T385M in patient 2, and this mutation was absent from her parents, brother and healthy controls. Lower panel (C) is taken from (21), with permission.

Patient 1 (P1), who was an 8-year-old female, was born as the third child of a Mexican mestizo family with a past history of an older sister who died before the age of 5 due to *Mycobacterium* and *Candida* infections after suffering from disseminated BCG disease from a BCG vaccine (20). The mutation causing the immunodeficiency in the offspring of the family was traced to the *IL12RB1* gene, which encodes the beta-1 chain of the IL-12/IL-23 receptor, with no expression of the protein in the affected homozygous children (Figure 1A) (20). The homozygous mutation in *IL12RB1* (1791+2 T>G) was determined at birth in P1 by genomic DNA sequencing of a cord blood sample. P1 received vaccines for hepatitis B, DPT, measles, mumps, and rubella without any adverse effects, but she did not receive the BCG vaccine and grew healthy without suffering infections. When P1 was 8 years old, she had a mild oral *C. albicans* infection, which was treated and controlled with topical nystatin. Following a second episode of oral candidiasis, she was treated with the IgG mouthwash procedure as described below.

Patient 2 (P2) was an 8-year-old girl diagnosed with a heterozygous *de novo* T385M missense mutation in the *STAT1* gene that was previously reported as a GOF mutation (Figure 1B) (21). P2 had recurrent and persistent oral *Candida* infections beginning at 8 months of age. She had received multiple drug treatments with fluconazole, nystatin, ketoconazole, and miconazole, with only partial improvements lasting from one to 3 weeks. At 8 years of age, after receiving unsuccessful treatments for oral candidiasis with nystatin and ketoconazole, she presented with severe oral candidiasis and was treated for 12 days with polyvalent IgG mouthwash.

### Treatment of Oral Candidiasis With Polyvalent IgG Mouthwash

P1 and P2 were treated on an ambulatory basis with human IV IgG (CSL Behring, PA, U.S.A) according to the following regimen. A 2-min mouthwash was performed with IV IgG solution containing 50 milligrams of IgG diluted in 10 mL of sterile water three times a day after meals and after thorough brushing of the teeth over a 12-day period. After overnight fasting, a daily specimen of early first mouthwash with 5 mL of sterile water was obtained in the morning prior to the brushing of the teeth. The sample was subsequently plated on Sabouraud agar media, and the number of *Candida* colonies was quantified as colony-forming units (CFU) at 24 h. On day 13, P1 continued to receive the IV IgG (as a mouthwash under the same scheme) and simultaneously started to receive 100,000 IU of nystatin suspension three times a day; IV IgG was administered topically until day 19, while nystatin was administered until day 23 (10 days of treatment with nystatin). P2 stopped the polyvalent human IgG schedule on day 13 when she was admitted to the hospital for treatment for a lower respiratory infection for which she received i.v. amoxicillin and caspofungin for 10 days.

## Results

### Opsonization of *C. albicans* Increases NADPH Oxidase Activity in Human Blood Leukocytes *in vitro*

**Figure 2:**
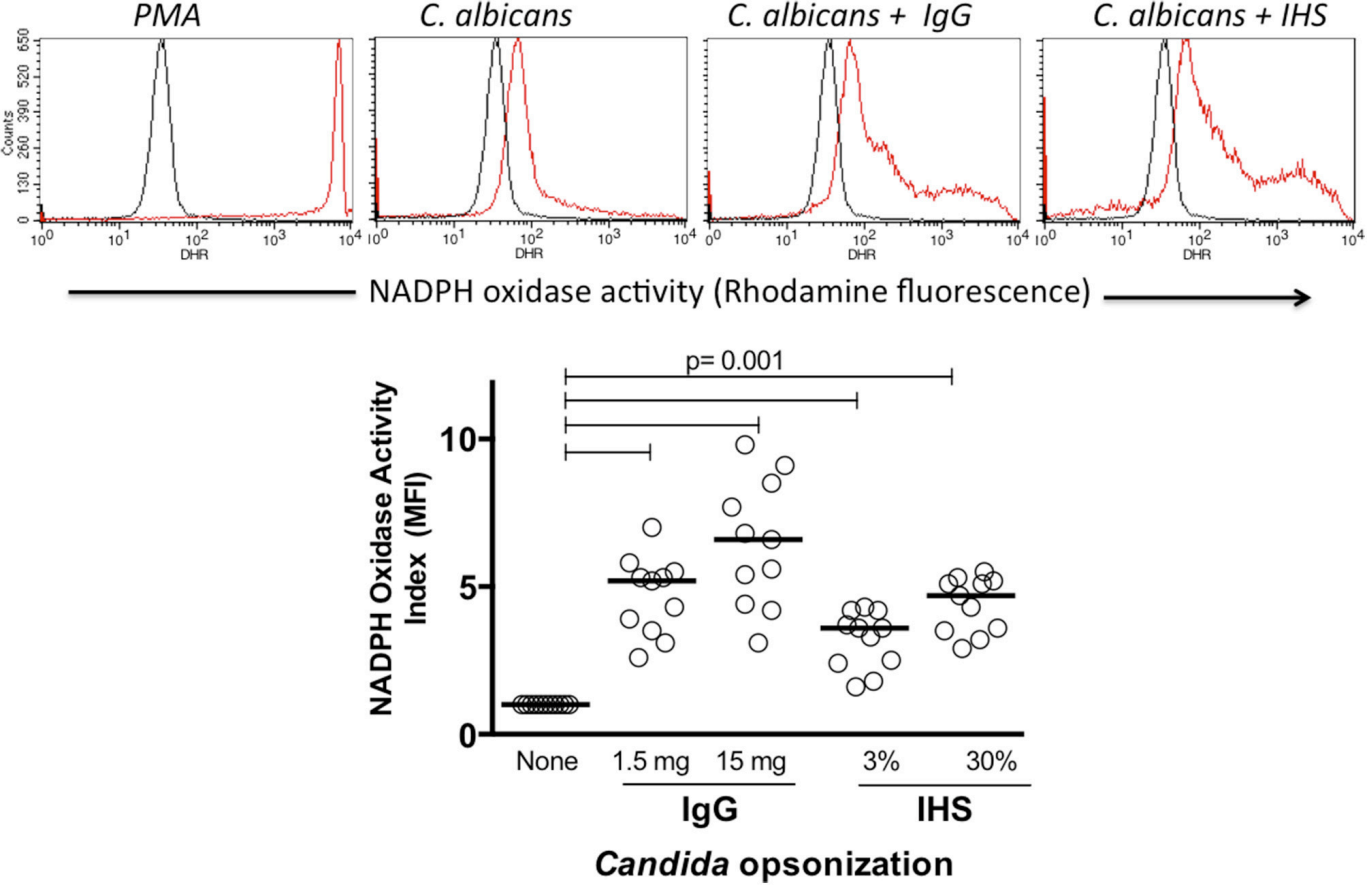
Upper histograms: example of NADPH oxidase assessment in blood leukocytes by flow cytometry (dihydrorhodamine assay). Black and red histograms indicate unstimulated and stimulated cells, respectively. The lower dot plot summarizes the NADPH oxidase stimulation indices of 11 healthy donors. IgG, commercial human polyvalent IgG IV IgG; IHS, immune human serum. Statistical test: Wilcoxon.

Because the production of oxygen metabolites is a main mechanism by which phagocytic cells kill intracellular microorganisms, we tested the capacity of Hk *C. albicans* with and without opsonization to induce NADPH oxidase activation *in vitro*. The rhodamine MFI, which was proportional to the NADPH oxidase activity, was augmented following the *in vitro* activation of leukocytes. In all experiments, the NADPH activity induced by PMA produced the highest rhodamine MFI as shown in Figure 2 in the upper panel, demonstrating the good cell viability and adequate performance of the test. Although both monocytes and neutrophils gated on FSC vs. SSC graphics showed NADPH oxidase activity in response to the *Candida albicans* stimulation (data not shown), we focused our analysis on the neutrophils. In all healthy donors tested, the NADPH activity was more enhanced in response to the opsonized *C. albicans* compared to that in response to the unopsonized preparations as shown in the upper panel of Figure 2. A comparative analysis of the NADPH oxidase activity under the activation conditions was performed using the MFI indices as described in the materials and methods. *C. albicans* opsonization using 1.5 or 15 mg/mL IgG significantly increased the NADPH activity (median of 5.2- and 6.8-fold with respect to unopsonized *Candida*) in all healthy donors tested. Similarly, the opsonization with HIS increased the NADPH activity by 3.7- and 4.4-fold when serum was used at 3 or 30%, respectively (Figure 2, lower panel) (*p* < 0.001). These results show that compared with unopsonized *C. albicans*, the antibody opsonization of *C. albicans* with polyvalent commercial human IgG or human serum clearly increases the NADPH oxidase activity of blood neutrophils in healthy human volunteers.

### Opsonized *C. albicans* Increases the Fungicidal Activity of Human Blood Leukocytes *in vitro*

In addition to assessing the NADPH oxidase activity produced by antibody-opsonized *C. albicans* and comparing with unopsonized *C. albicans*, we investigated the actual killing activity of the fungus by blood leukocytes from healthy donors to determine whether opsonization helped to eliminate *Candida*.

The assessment of *C. albicans* killing using a whole blood assay has the advantage of preserving human phagocytes in their media under a more physiological condition without introducing the stress of cell purification. The experimental conditions of the assays (after the preliminary experiments) were established with a 1-h infection of whole blood with a fixed amount of *C. albicans* yeast in a shaker, followed by hypotonic cell lysis and seeding of biological material; the CFU were assessed at 24 h.

**Figure 3:**
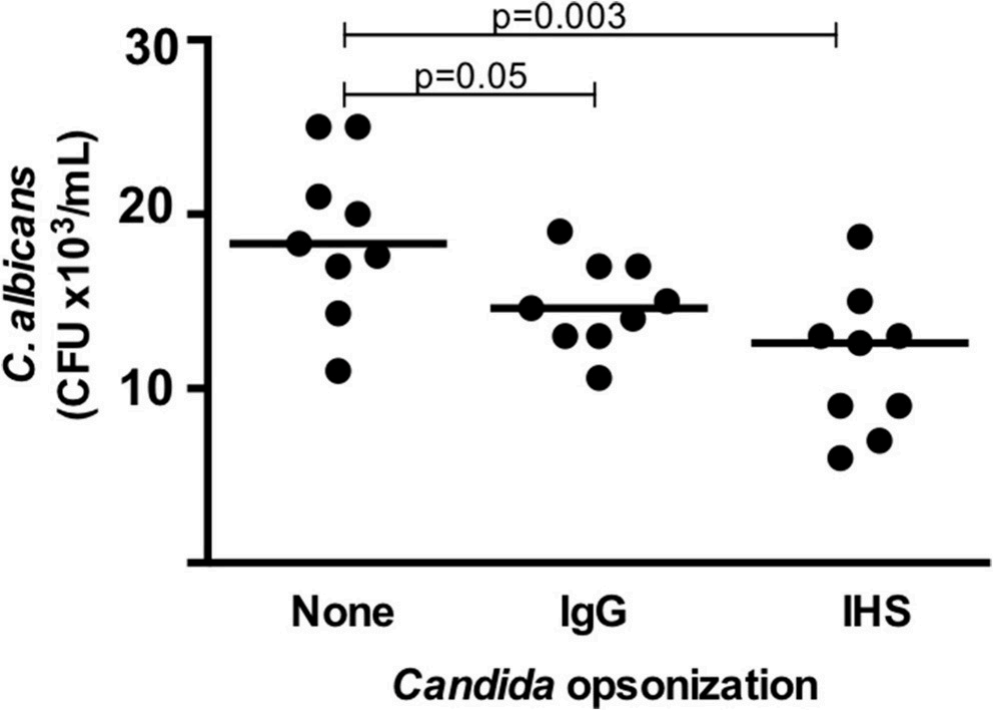
Fungicidal activity targeting *C. albicans* determined in a whole blood assay (at *t* = 1 h) as assessed by CFU counts in 9 healthy donors. Each dot represents a donor, and the horizontal bars represent the medians. IgG, IV IgG commercial human polyvalent IgG; IHS, immune human serum; Statistical test: Wilcoxon.

The killing activity performed by cells in whole blood (putatively by neutrophils and monocytes) against unopsonized *Candida albicans* was variable among the samples from the 9 healthy donors tested, resulting in a median CFU of 18.2 × 10^3^ in the experiments (range, 11–26 × 10^3^ CFU). When *Candida* was opsonized with IV IgG and tested in the experiments, the number of CFUs at the endpoint decreased in all donors (median, 14.6 × 10^3^; range, 10.5–19 × 10^3^), and when *Candida* was opsonized with IHS, the number of CFUs decreased further (median, 12.8 × 10^3^; range, 7–18.5 × 10^3^) (*p* = 0.05 and 0.003, respectively, Figure 3). We also tested *C. albicans* killing using a pool of commercial sera obtained from blood type AB positive donors for opsonization and found an adjuvant effect that produced an enhanced killing capacity between that reached with IV IgG and that with immune human sera (data not shown). These significant decreases in the CFU compared with unopsonized *Candida* and *Candida* opsonized with IV IgG or immune sera indicate that there was an increase in the killing activity of blood leukocytes in these experiments, which was likely enhanced by the antibodies.

### Daily Treatment With IgG Mouthwash Drastically Reduced Oral Candida Infection in Two Immunodeficient Patients

**Figure 4:**
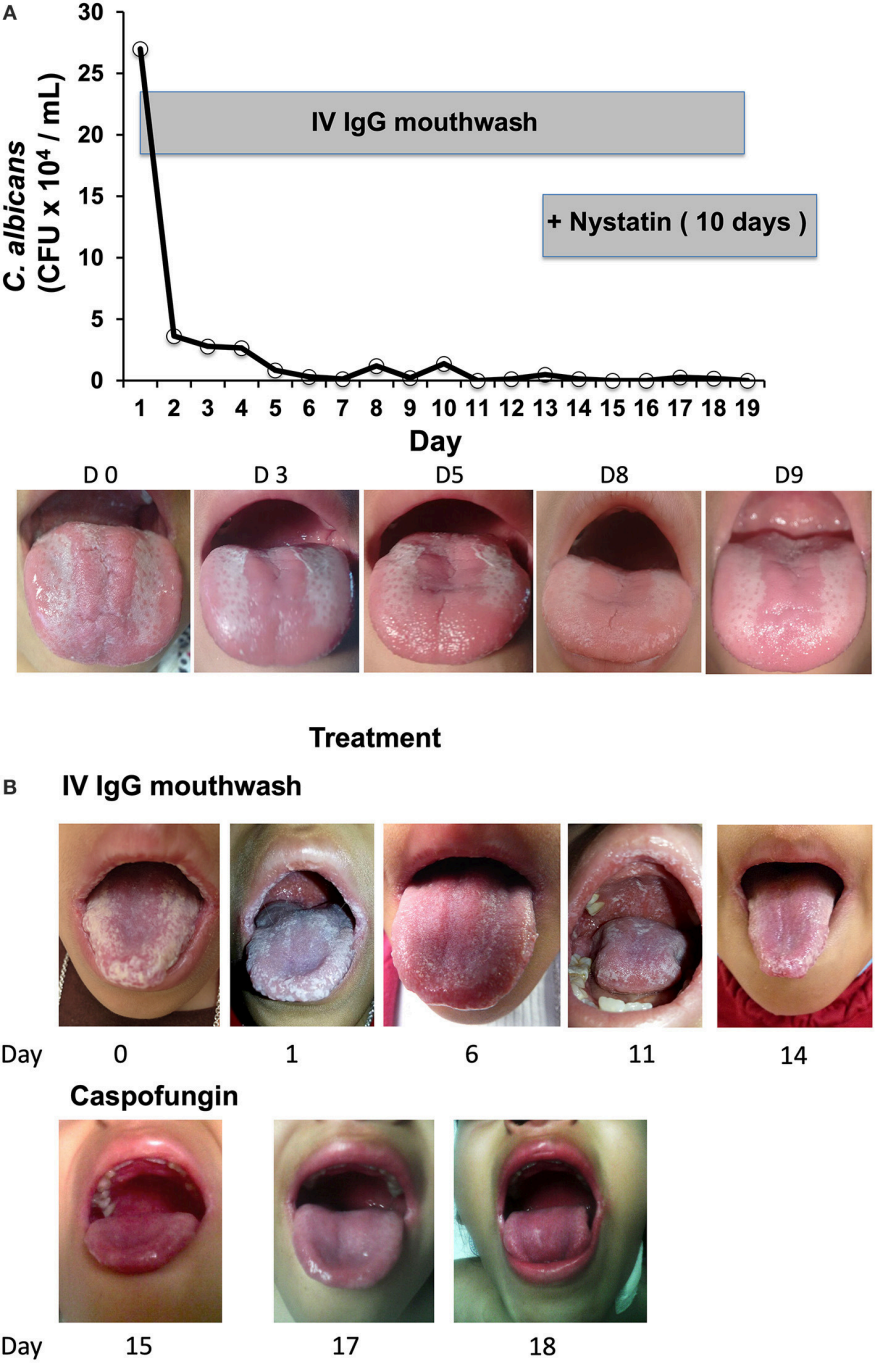
**(A)** Daily microbiological assessment of *Candida* CFUs in the mouth of Patient 1 (with a mutation in the *IL12RB1* gene) in treatment during 19 days with IV IgG mouthwash (plot), and representative images of infection evolution. **(B)** Patient 2, who had a T385M GOF mutation in *STAT1*, was treated for 13 days with IgGmw for CMC. Images of the treatment with only IV IgG (upper), and only with i.v. caspofungin plus amoxicillin, which was started on day 15 (lower) are shown.

The results of the *in vitro* experiments with the healthy volunteer blood samples showed that *C. albicans* opsonization with IV IgG augmented the NADPH and killing activity of phagocytes. On the basis of these results, we tested *in vivo* the effects of the opsonization of *C. albicans* with IV IgG in two patients with oral candidiasis and primary immunodeficiency (PID) and observed favorable results. The first patient, P1, who had an IL-12Rβ1 deficiency and *C. albicans* oral infection, exhibited a positive response to the IgG mouthwash as the number of CFUs of *C. albicans* in the first daily mouthwash (collected in the morning before eating and brushing teeth) dramatically decreased by 10-fold after only 2 days of treatment (Figure 4A, upper panel). The *C. albicans* CFU counts continued to decrease over the following days, and on days 11, 15, 16, and 19, the CFUs were null (IgG treatment lasted 19 days for P1). The mouth images of P1 (Figure 4A, lower panel) showed a clear improvement as follows: at the beginning of treatment (day 0), there was intense erythematous candidiasis in the tongue and pseudomembranous candidiasis with white plaques on the tongue and oropharynx; by day 5, the pseudomembranous candidiasis disappeared, and by days 8 and 9, the pseudomembranous candidiasis on the tongue and oropharynx disappeared, which is consistent with the results of the cultures as shown in the plot. Complementary treatment with oral nystatin suspension for 10 days starting on day 13 completely removed the clinical signs of infection, and the patient stopped all treatments. P1 has been free of oral candidiasis for more than 1 year and a half as of the submission of this paper.

P2 has a T385M heterozygous *STAT1* GOF mutation (21), and her cells did not produce IL-17 upon the PMA+ionomycin stimulation (Figure 1, lower panels B and C); furthermore, she had CMC refractory to treatment. P2 first presented with severe oral candidiasis affecting the tongue, palate, and lips. On day 0, P2 presented with thick, white crude plaques on the dorsal surface of the tongue, lips, palate, and oropharynges (Figure 4B, first picture). P2 started receiving treatment with only the IV IgG mouthwash under the same schedule as P1. The clearance of *Candida* infection in her mouth improved as shown in the daily images. For logistical reasons, it was not possible to follow the control of infection by microbiologic cultures as was performed for P1. P2 showed a clear improvement in her mouth following the IV IgG mouthwash treatment, and after 1 week, the appearance of tongue, palate, and lips was substantially clearer after receiving the IV IgG (Figure 4B, day 6) with the resolution of halitosis. The improvement in the oral lesions in P2 allowed her to begin eating better after 1 week; before treatment, eating was painful, and very difficult to perform. On day 14, when the patient was admitted to the hospital for treatment for a lower airway infection, the mouth *Candida* infection had improved by ~70% compared with that at the beginning of the treatment as evidenced by images. An oral water wash obtained on day 13 was cultured, and *C. albicans* was identified resistant to voriconazole. In addition, an oral coinfection with *Streptococcus* sp. was also found in the culture. During the hospitalization, P2 received intravenous amoxicillin and caspofungin, and after 3 days of this treatment, her mouth was free of *Candida* as evidenced by negative culture. After 1 year and a half receiving the IV IgG mouthwash treatment, P2 only had mild episodes of oral candidiasis, which responded to nystatin, and subsequently, she ceased having severe oral candidiasis.

## Discussion

In this study, we first demonstrated that *in vitro* opsonization of *C. albicans* with IV IgG increased the NADPH oxidase activity of neutrophils and enhanced the killing of *C. albicans* in blood samples from healthy volunteers. On the basis of these results, we tested the *in vivo* effect of *Candida* sp. opsonization on its elimination and evaluated the use of IV IgG mouthwash for the treatment of oral candidiasis in two patients with PID and deficient IL-17 production and observed positive results with a significant reduction in infection in both patients.

Several previous studies published by other investigators have shown the enhancing role of antibody opsonization in *C. albicans* killing by blood leukocytes. Bliss et al. (22) challenged *in vitro* peripheral blood human mononuclear cells with serum opsonized *C. albicans* and found a fungicidal effect mediated by specific antibodies that was dependent on monocytes. The antibody-dependent mechanism of *C. albicans* killing by neutrophils uses Fc-γ receptors, protein kinase C (PKC), and reactive oxygen metabolites, while the antibody-independent mechanism depends on complement 3 receptor (CR3), PI3K and CARD9 (17). Since we observed in our *in vitro* experiments a significant improvement in NADPH oxidase activity and killing by IV IgG opsonization, we suggest that the main mechanism of the killing is mediated by Fc-γ receptors, PKC, NADPH oxidase, and oxygen metabolites.

The role of antibodies in the control of experimental candidiasis *in vivo* has been demonstrated in several studies. For example, Torosantucci et al. (11) showed that the IgG2b monoclonal antibody anti-beta-glucan of *C. albicans* protected mice from kidney infection, and the monoclonal antibody inhibited the growth of cultures of *C. albicans*. In animal models of immunization, antibodies have been shown to play a role in reducing or protecting against systemic *Candida* infection and promoting survival in challenged animals (11–13) [reviewed in (23)]. In humans and *in vivo*, bovine anti-*Candida* antibodies have been shown to aid in preventing oral candidiasis in recipients of bone marrow transplants (24). Interestingly in a recent study with 71 patients with candidemia, it was shown that high titers of anti-MP65 antibodies (recognizing a cell wall component of *Candida*) are related to surviving to the severe disseminated infections (14). Other authors have found that anti-*Candida* IgY (chicken egg-yolk immunoglobulin) reduces the *Candida* lingual load in mice orally infected with *C. albicans*, and the antibodies diminish the dissemination of the fungus to the intestines, kidneys, and lungs (25).

To the best of our knowledge, the two patients with PID (with proven mutations in the genes *IL12RB1* and *STAT1*) and oral candidiasis presented in this study are the first to demonstrate successful therapy with polyvalent human IgG by mouthwash. Chronic mucocutaneous candidiasis (CMC) is a persistent or recurrent infection affecting the skin, nails, and oral, or genital mucosa and is caused by *Candida* spp, mostly by *C. albicans* (26). CMC susceptibility has been related to defects in T cell responses, mainly in IL-17-producing cells, such as Th17 and γ/δ T cells; individuals with mutations affecting the production of IL-17 and the proportion of Th17 cells (such as mutations in the genes *IL17RA, IL17RC, IF17F, ACT 1, STAT3, AIRE, RORC, CARD9, IL12B*, and *IL12RB1*, or GOF mutations in *STAT1*) suffer from CMC and other infections with variable severity [reviewed in (6, 7)]. In a study published in 2014, *Candida* infections (mainly oropharyngeal) were found in 43 of 151 (28%) patients with mutations in the *IL12RB1* gene (27), and the fungal infection was recurrent or persistent in 74% of those patients despite oral or intravenous antifungal treatments (mainly with nystatin or fluconazole). The evidence presented in that paper suggests that since mucocutaneous candidiasis in those patients could be disseminated to the esophagus or other organs and tissues of the body, controlling oral candidiasis is important for preventing systemic spread. The P1, who had a mutation in the *IL12RB1* gene, manifested an oral *Candida* infection as the only infection as a consequence of her PID. The treatment with only the IV IgG mouthwash cleared the infection almost completely within 13 days, and the infection was completely eliminated by additional oral nystatin as demonstrated by the clinical and microbiological assessments.

In patient P2, who manifested a GOF mutation in the *STAT1* gene and severe CMC since the age of 8 months with very poor responses to many pharmacological treatments, the severe oral *Candida* infection (resistant to voriconazole) was cleared to a great extent with only the IV IgG mouthwash treatment. In addition, at the hospital, the mucosal infection responded to intravenous treatment with amoxicillin plus caspofungin with rapid clearing of *Candida*. This response is remarkable compared to the less favorable treatment responses reported in the literature. In a recent paper reviewing the clinical characteristics of 274 patients with GOF *STAT1* mutations, candidiasis was found in 98% of patients, and mucocutaneous candidiasis was the most frequently localized form of candidiasis, while 10% of the patients also had a disseminated variety. In addition, candidal drug resistance was found in 78 of 202 patients (38%) treated with long-term anti-fungal therapy (with the “azole” antifungal drugs fluconazole, itraconazole, posaconazole, or voriconazole) (28), thus demonstrating the difficulties in the treatment of these patients. Mossner et al. used another strategy for the treatment of CMC in an adult patient with severe drug-resistant oral candidiasis since childhood and the ST*AT1* GOF mutation p.R274Q. The patient was treated with Ruxolitinib, which is a tyrokinase inhibitor of Jak phosphorylation (thus indirectly preventing Stat-1 hyperphosphorylation in the patient) for 6 months; following this treatment, a restitution of IL-17 production was observed with general systemic health improvement but only a partial improvement in oral candidiasis (29). Thus, in this context, the treatments of oral candidiasis with polyvalent human IgG mouthwash offer promising and encouraging therapeutic options for PID patients afflicted with these clinically perplexing candidal infections.

Two apparent advantages of the IgG mouthwash regimen for mucosal candidiasis is that avoids the drug-resistance and that are non-invasive. Frequently, conventional drug treatments for mucosal candidiasis select resistant strains and, thus, are no longer effective in fungal elimination and can occasionally have toxic effects if taken over long periods of time, producing additional deleterious effects. Local treatment for mucosal candidiasis with human polyvalent IgG could be an optional adjuvant treatment in these cases; choosing IV IgG for the opsonization of *Candida* by repeated mouthwash has the advantage of giving controlled amounts of antibodies in a non-invasive form of topical treatment. Since IV IgG is obtained from a large number of healthy donors, it could contain specific antibodies able to recognize *Candida* antigens.

In conclusion, in this study, we show that the treatment of oral candidiasis with polyvalent human IgG mouthwash helped eliminate the infection in two patients with an IL-12Rβ1 deficiency and a ST*AT1* GOF mutation. This treatment strategy could be used for the treatment of other immunocompromised patients with oral candidiasis, who frequently are infected with drug-resistant strains, or as immuno-prophylaxis in patients who are at risk of developing *Candida* infection, e.g., recipients of bone marrow transplants or patients with cancer undergoing radiotherapy or chemotherapy. The limitation of our study is that the treatment was evaluated in only two patients. More research is necessary with additional patients and different sources of antibodies and dosage regimens to more completely understand the mechanisms involved in *Candida* elimination and their clinical translation to therapy for patients afflicted with these clinically relevant infections.

## Ethics Statement

This study was conducted according to the principles expressed in the last version of the Declaration of Helsinki in 2013. The protocol was approved by the Committee of Ethics in Research of the Instituto Nacional de Ciencias Médicas y Nutrición Salvador Zubirán, and all participants or the legal guardians of the participants under the age of 18 provided written informed consent.

## Author Contributions

SP-S conceived the design of the study, performed the experiments and wrote the paper. JM-L provided medical care and follow-up to the patients and contributed to the design of the mouthwash protocol. YG determined the mutation in P2 and contributed to the writing of the paper. MTH and MLV-A performed the experiments. JL-F provided medical care to the patients and the medical information of the patients. JB contributed to the design of the study and to the writing of the paper, and MT coordinated the work and wrote the paper.

### Conflict of Interest Statement

The authors declare that the research was conducted in the absence of any commercial or financial relationships that could be construed as a potential conflict of interest.

## References

[1] Kim J Sudbery P. Candida albicans, a major human fungal pathogen. J Microbiol. (2011) 49:171–7. 10.1007/s12275-011-1064-7 21538235

[2] Cheng SC Joosten LA Kullberg BJ Netea MG. Interplay between Candida albicans and the mammalian innate host defense. Infect Immun. (2012) 80:1304–13. 10.1128/IAI.06146-11 22252867 PMC3318407

[3] Srivastava V Singla RK Dubey AK. Emerging virulence, drug resistance and future anti-fungal drugs for candida pathogens. Curr Top Med Chem. (2018) 18:759–78. 10.2174/1568026618666180528121707 29807516

[4] Whaley SG Berkow EL Rybak JM Nishimoto AT Barker KS Rogers PD. Azole antifungal resistance in candida albicans and emerging non-albicans candida species. Front Microbiol. (2016) 7:2173. 10.3389/fmicb.2016.02173 28127295 PMC5226953

[5] Richardson JP Moyes DL. Adaptive immune responses to Candida albicans infection. Virulence (2015) 6:327–37. 10.1080/21505594.2015.1004977 25607781 PMC4601188

[6] Okada S Puel A Casanova JL Kobayashi M. Chronic mucocutaneous candidiasis disease associated with inborn errors of IL-17 immunity. Clin Transl Immunol. (2016) 5:e114. 10.1038/cti.2016.71 28090315 PMC5192062

[7] Puel A Cypowyj S Marodi L Abel L Picard C Casanova JL. Inborn errors of human IL-17 immunity underlie chronic mucocutaneous candidiasis. Curr Opin Allergy Clin Immunol. (2012) 12:616–22. 10.1097/ACI.0b013e328358cc0b 23026768 PMC3538358

[8] Cabezas J Albaina O Montanez D Sevilla MJ Moragues MD Ponton J. Potential of anti-Candida antibodies in immunoprophylaxis. Immunotherapy (2010) 2:171–83. 10.2217/imt.09.76 20635926

[9] Moragues MD Omaetxebarria MJ Elguezabal N Sevilla MJ Conti S Polonelli L. A monoclonal antibody directed against a Candida albicans cell wall mannoprotein exerts three anti-*C. albicans* activities. Infect Immun. (2003) 71:5273–9. 10.1128/IAI.71.9.5273-5279.2003 12933874 PMC187351

[10] Richardson M Rautemaa R. How the host fights against Candida infections. Front Biosci. (2009) 1:246–57. 10.2741/3533 19482700

[11] Torosantucci A Chiani P Bromuro C De Bernardis F Palma AS Liu Y Cassone: protection by anti-beta-glucan antibodies is associated with restricted beta-1,3 glucan binding specificity and inhibition of fungal growth and adherence. PLoS ONE (2009) 4:e5392 10.1371/journal.pone.0005392 19399183 PMC2670538

[12] Han Y Cutler JE. Antibody response that protects against disseminated candidiasis. Infect Immun. (1995) 63:2714–9. 7790089 10.1128/iai.63.7.2714-2719.1995 PMC173363

[13] Torosantucci A Bromuro C Chiani P De Bernardis F Berti F Galli C. A novel glyco-conjugate vaccine against fungal pathogens. J Exp Med. (2005) 202:597–606. 10.1084/jem.20050749 16147975 PMC2212864

[14] Torosantucci A Tumbarello M Bromuro C Chiani P Posteraro B Sanguinetti M. Antibodies against a beta-glucan-protein complex of Candida albicans and its potential as indicator of protective immunity in candidemic patients. Sci Rep. (2017) 7:2722. 10.1038/s41598-017-02977-6 28578431 PMC5457410

[15] van Kessel KP Bestebroer J van Strijp JA. Neutrophil-mediated phagocytosis of *Staphylococcus aureus*. Front Immunol. (2014) 5:467. 10.3389/fimmu.2014.00467 25309547 PMC4176147

[16] Lehrer RI Cline MJ. Interaction of Candida albicans with human leukocytes and serum. J Bacteriol. (1969) 98:996–1004. 4182532 10.1128/jb.98.3.996-1004.1969 PMC315286

[17] Gazendam RP van Hamme JL Tool AT van Houdt M Verkuijlen PJ Herbst M. Two independent killing mechanisms of Candida albicans by human neutrophils: evidence from innate immunity defects. Blood (2014) 124:590–7. 10.1182/blood-2014-01-551473 24948657

[18] Richardson MP Ayliffe MJ Helbert M Davies EG. A simple flow cytometry assay using dihydrorhodamine for the measurement of the neutrophil respiratory burst in whole blood: comparison with the quantitative nitrobluetetrazolium test. J Immunol Methods (1998) 219:187–93. 10.1016/S0022-1759(98)00136-7 9831400

[19] Wallis RS Palaci M Vinhas S Hise AG Ribeiro FC Landen K. A whole blood bactericidal assay for tuberculosis. J Infect Dis. (2001) 183:1300–3. 10.1086/319679 11262217

[20] Pedraza-Sanchez S Herrera-Barrios MT Aldana-Vergara R Neumann-Ordonez M Gonzalez-Hernandez Y Sada-Diaz E. Bacille calmette-guerin infection and disease with fatal outcome associated with a point mutation in the interleukin-12/interleukin-23 receptor beta-1 chain in two Mexican families. Int J Infect Dis. (2010) 14(Suppl. 3):e256–60. 10.1016/j.ijid.2009.11.005 20171917

[21] Pedraza-Sanchez S Lezana-Fernandez JL Gonzalez Y Martinez-Robles L Ventura-Ayala ML Sadowinski-Pine S. Disseminated tuberculosis and chronic mucocutaneous candidiasis in a patient with a gain-of-function mutation in signal transduction and activator of transcription 1. Front Immunol. (2017) 8:1651. 10.3389/fimmu.2017.01651 29270166 PMC5723642

[22] Bliss JM Laforce-Nesbitt SS. Toxicity to Candida albicans mediated by human serum and peripheral blood mononuclear cells. FEMS Immunol Med Microbiol. (2006) 46:452–60. 10.1111/j.1574-695X.2006.00063.x 16553821

[23] Wang XJ Sui X Yan L Wang Y Cao YB Jiang YY. Vaccines in the treatment of invasive candidiasis. Virulence (2015) 6:309–15. 10.4161/21505594.2014.983015 25559739 PMC4601158

[24] Tollemar J Gross N Dolgiras N Jarstrand C Ringden O Hammarstrom L. Fungal prophylaxis by reduction of fungal colonization by oral administration of bovine anti-Candida antibodies in bone marrow transplant recipients. Bone Marrow Transplant. (1999) 23:283–90. 10.1038/sj.bmt.1701560 10084261

[25] Ibrahimel SM Rahman AK Isoda R Umeda K Van Sa N Kodama Y *In vitro* and *in vivo* effectiveness of egg yolk antibody against Candida albicans (anti-CA IgY). Vaccine (2008) 26:2073–80. 10.1016/j.vaccine.2008.02.046 18375022

[26] Kirkpatrick CH. Chronic mucocutaneous candidiasis. Pediatr Infect Dis J. (2001) 20:197–206. 10.1097/00006454-200102000-00017 11224843

[27] Ouederni M Sanal O Ikinciogullari A Tezcan I Dogu F Sologuren I. Clinical features of Candidiasis in patients with inherited interleukin 12 receptor beta1 deficiency. Clin Infect Dis. (2014) 58:204–13. 10.1093/cid/cit722 24186907 PMC3871796

[28] Toubiana J Okada S Hiller J Oleastro M Lagos Gomez M Aldave Becerra JC. Heterozygous STAT1 gain-of-function mutations underlie an unexpectedly broad clinical phenotype. Blood (2016) 127:3154–64. 10.1182/blood-2015-11-679902 27114460 PMC4920021

[29] Mossner R Diering N Bader O Forkel S Overbeck T Gross U. Ruxolitinib induces interleukin 17 and ameliorates chronic mucocutaneous candidiasis caused by STAT1 gain-of-function mutation. Clin Infect Dis. (2016) 62:951–3. 10.1093/cid/ciw020 26787170

